# Early Diagnosis of Brain Metastases Using a Biofluids-Metabolomics Approach in Mice

**DOI:** 10.7150/thno.16538

**Published:** 2016-09-12

**Authors:** James R. Larkin, Alex M. Dickens, Timothy D. W. Claridge, Claire Bristow, Kleopatra Andreou, Daniel C. Anthony, Nicola R. Sibson

**Affiliations:** 1 Cancer Research UK and Medical Research Council Oxford Institute for Radiation Oncology, Department of Oncology, University of Oxford, Oxford, UK;; 2 Department of Pharmacology, University of Oxford, Oxford, UK;; 3 Department of Chemistry, University of Oxford, Oxford, UK.

**Keywords:** Brain metastasis, diagnosis, metabolomics, NMR, animal model.

## Abstract

Over 20% of cancer patients will develop brain metastases. Prognosis is currently extremely poor, largely owing to late-stage diagnosis. We hypothesized that biofluid metabolomics could detect tumours at the micrometastatic stage, prior to the current clinical gold-standard of blood-brain barrier breakdown.

Metastatic mammary carcinoma cells (4T1-GFP) were injected into BALB/c mice via intracerebral, intracardiac or intravenous routes to induce differing cerebral and systemic tumour burdens. B16F10 melanoma and MDA231BR-GFP human breast carcinoma cells were used for additional modelling. Urine metabolite composition was analysed by ^1^H NMR spectroscopy. Statistical pattern recognition and modelling was applied to identify differences or commonalities indicative of brain metastasis burden.

Significant metabolic profile separations were found between control cohorts and animals with tumour burdens at all time-points for the intracerebral 4T1-GFP time-course. Models became stronger, with higher sensitivity and specificity, as the time-course progressed indicating a more severe tumour burden. Sensitivity and specificity for predicting a blinded testing set were 0.89 and 0.82, respectively, at day 5, both rising to 1.00 at day 35. Significant separations were also found between control and all 4T1-GFP injected mice irrespective of route. Likewise, significant separations were observed in B16F10 and MDA231BR-GFP cell line models. Metabolites underpinning each separation were identified**. **

These findings demonstrate that brain metastases can be diagnosed in an animal model based on urinary metabolomics from micrometastatic stages. Furthermore, it is possible to separate differing systemic and CNS tumour burdens, suggesting a metabolite fingerprint specific to brain metastasis. This method has strong potential for clinical translation.

## Introduction

Metastasis is a leading cause of cancer mortality and remains a significant clinical hurdle. Approximately 20-40% of cancer patients will develop brain metastases 1,2 with lung, breast and melanoma primary tumours accounting for 67 to 80% of all cases 3. Critically, brain metastases present a particularly difficult clinical challenge owing to the nature of the blood-brain barrier (BBB), which prevents both diagnosis of tumours and their treatment with most systemically active therapeutic agents in the early stages when therapy could be maximally effective.

Brain metastasis prognosis is extremely poor 4, likely reflecting the late stage of diagnosis 5,6. The current gold standard for diagnosis is gadolinium-enhanced MRI 7-9, which is dependent on the relatively late stage event of BBB breakdown. There is an urgent need, therefore, to develop new and sensitive methods for earlier detection of brain metastasis. It is hypothesized that, if brain metastasis could be detected earlier, treatments could be better directed and prognosis improved, whilst harmful and unnecessary treatment of at-risk cancer patients without brain metastases could be avoided 10.

Spectroscopic analysis of biofluids, using high resolution ^1^H NMR, involves the acquisition of spectra followed by multivariate statistical pattern recognition analysis. This powerful approach requires no *a priori* knowledge, but rather identifies metabolites solely from correlated variation between treatment groups 11. Thus, rather than requiring single biomarkers, distinct patterns of metabolites whose variation as a whole is characteristic of a disease can be identified. This may be a particularly useful technique to study brain metastases as the multiple cell types present in the brain microglia, astrocytes and neurons), coupled with unique anatomical features such as the blood-brain barrier, mean that the brain often exhibits a differing response to challenges than the rest of the body. It is likely that the brain's response to the tumour is thus just as unique as its physiology and anatomy, which may make a tumour signature unique.

Using this metabolomics approach, we recently demonstrated that it was possible to distinguish between relapsing remitting and secondary progressive multiple sclerosis patients in a predictive fashion 12. Others have recently shown that there is scope to predict patients with systemic micrometastatic disease using serum metabolomics 13. Our primary aim here, therefore, was to determine whether a biofluid metabolomics approach could enable differentiation between mice specifically with brain metastases and those without. Subsequently, we asked whether this approach is sensitive to differing systemic and CNS burdens of metastasis, and whether distinct biomarkers for systemic and CNS metastases could be identified.

## Materials and Methods

### Animal models

Female BALB/c mice (6-7 weeks, Charles River, UK, n=50) were housed under a standard 12h light 12h dark cycle and with access to standard chow and water ad libitum. Female mice were chosen to recapitulate brain metastasis from breast tumours as primary breast tumours predominantly occur in the female population. For surgery, they were anaesthetized with isoflurane (1.5-2.0%) in 30% O_2_ with 70% N_2_O, placed in a stereotaxic frame and a burr hole drilled above the injection site (bregma: +0.5mm; left 1.9mm; depth 2.9mm). Using a glass microcannula (tip diameter *ca.* 75µm), 5x10^3^ 4T1-GFP cells (a mouse mammary carcinoma cell line) in 0.5µL PBS or vehicle alone were injected into the left striatum over a period of 5 min. The microcannula was left in place for 5 min, raised by 0.5mm and left for a further 2 min before complete removal. Urine was collected by handling the mice over a clean and impermeable surface, both before injection and at days 5, 10, 21 and 35 after injection then stored at -80°C. Urine samples were collected early in the morning but if the bladder was found to be empty (approximately 25% of mice), samples were collected later in the day. The 4T1 parent cell line was originally obtained from the ATCC (CRL-2539) and was stably transfected to express EFGP 14. As a mouse cell line, there is no publicly available short tandem repeat (STR) profile for the cells thus they have not been externally authenticated.

Two further groups of BALB/c mice (n=20) were injected either intracardially or intravenously with 1x10^5^ 4T1-GFP cells in 100µL PBS or vehicle alone to investigate systemic tumor contribution to the metabolomic models; injection via different routes gives rise to differing systemic and CNS metastatic burdens 15. Urine was collected pre- and 10 days post-injection. For intracardiac injections, animals were anaesthetized with isoflurane (1.5-2.0%) in 100% O_2_, and the hair covering the thoracic cavity was removed by clipping and depilatory cream. Ultrasound imaging was used to guide injection of cells into the left ventricle of the heart. Animals were allowed to recover in a heated chamber 14. Intravenous injections were made in awake restrained animals through a lateral tail vein.

In addition to the 4T1-GFP model, two further experimental models were used: (i) metastatic human breast carcinoma cells (MDA231BR-GFP) injected intracerebrally in SCID mice; and (ii) metastatic mouse melanoma cells (B16F10) injected intracerebrally in syngeneic C57BL/6 mice (Charles River, UK; 5x10^3^ cells in 0.5µL PBS for both). In each case, urine samples were collected 10 days after tumour cell injection (n=5 and n=6 for MDA231BR-GFP and B16F10 models, respectively). PBS-injected (n=6 and n=5, respectively) and naïve mice (n=5 for each strain) were included as controls. A summary of animal models used is provided in Table 1. All animal procedures were approved by the UK Home Office.

Following sacrifice, animals were transcardially perfused with heparinized saline followed by 50mL PLP_light_ (periodate-lysine-paraformaldehyde with 0.025% w/v glutaraldehyde) 16. Brains were cryoprotected, frozen, sectioned (10µm thickness), quenched with 1% (v/v) hydrogen peroxide (30% w/v) in methanol and blocked with 1% normal rabbit serum in PBS for 1h. Immunohistochemistry was performed on brain sections bearing 4T1-GFP or MDA231BR-GFP tumours with a chicken anti-GFP antibody (Abcam, UK; 1:1000, 4°C, overnight), corresponding biotinylated secondary antibodies and visualized using 3,3'-diaminobenzidine (DAB) with cresyl violet counterstain. Brain sections bearing B16F10 tumours were only counterstained as they lack GFP but express high concentrations of melanin making them naturally black. Slides were scanned with a ScanScope slide scanner and tumours were circumscribed. Tumour volume was estimated by multiplying circumscribed tumour area by slice thickness.

### NMR spectroscopy

Urine (50µL) was defrosted on ice and mixed with NMR buffer (0.24M sodium phosphate, pH 7.4, 0.1% sodium azide, 0.8% sodium chloride in D_2_O containing 1mM TSP (3-trimethylsilyl-1-[2,2,3,3,-^2^H_4_] propionate); 550µL). ^1^H NMR spectra were acquired for each sample at 700MHz (Bruker Avance III spectrometer equipped with a 1H TCI cryoprobe) using a NOESY pre-saturation sequence with a mixing time of 10ms and solvent-presaturation during the relaxation delay (2s). Two-dimensional correlation spectroscopy (COSY) ^1^H NMR spectra were acquired from a single sample within each group to assist with metabolite identification. COSY spectra were acquired with 1.5s solvent presaturation, a spectral width of 10ppm (7002Hz), and 16 or 32 transients per t1 increment for 256 increments. All NMR experiments were conducted at 293K.

All processing was conducted in Matlab. Spectra were imported and automatically phased 17. Spectra with gross distortions or phasing anomalies were excluded. Spectra were baseline corrected with a 3^rd^ order polynomial fitted to regions without peaks 18 and aligned to TSP before being unit scaled to the summed integral excluding water and TSP. Spectra were refined by non-linear warping to correct peak shifts arising from differing pH, ionic strength etc. 19. Spectra were sub-divided into 0.01ppm regions (δ = start of integral region) from 0.2 to 9.6ppm and integrated. The variable water peak (4.7 to 5.0ppm) and urea peak (5.70 to 5.95ppm) were excluded along with a contaminant methanol peak (3.35 to 3.38ppm). Thus, modelling was conducted with 882 variables for each sample.

### Statistical analysis

**Figure 1:**
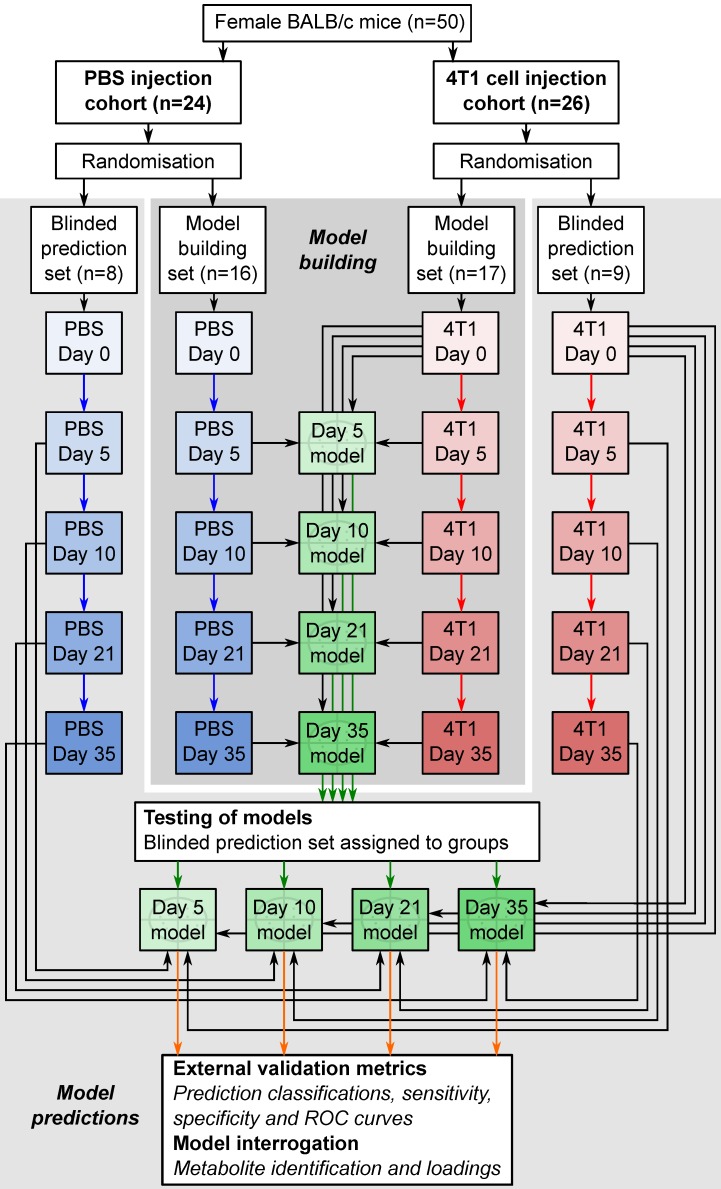
** Flowchart of study design.** The flowchart shows study design for the intracerebral 4T1-GFP brain metastasis model. Both 4T1-GFP cell-injected and PBS-injected cohorts were split into two groups, one for model building and one reserved as a supplemental blinded testing set. Each model was then constructed comparing 4T1-GFP animals from a particular day with the control set comprising PBS-injected animals from the same day as well as the naïve 4T1-GFP animal samples from before injection. The reserved testing set is used in a blinded fashion to test model predictive ability.

Orthogonal partial least squares discriminant analysis (OPLS-DA) modelling was conducted using SIMCA 13.0 (Umetrics, Sweden) to produce models which maximally separated groups of spectra. For the 4T1-GFP time-course, four models were constructed to separate samples from mice at day 5, 10, 21 or 35 from their respective control cohorts. The control cohorts included samples from the 4T1-GFP injected mice before they were injected with cells on day 0, together with samples from age and time-point matched mice injected with PBS (see study schematic in Figure 1). This combined control cohort controls for differences between batches of mice, as well as the effect of the injection procedure itself; any remaining differences are attributable to tumour presence. Analogously, models for alternate cell lines were designed to separate tumour-injected animals from a combined naïve and PBS-injected control cohort.

All data were centred and scaled using Pareto variance in order to suppress the noise present. To determine the potential predictive value of the models, the q^2^ value for each model was calculated. The q^2^ of a model is derived from a step-wise removal of a fraction of samples and a prediction of the group membership of the removed samples using a model built with the remaining samples. A q^2^>0 indicates a predictive model, whilst a q^2^>0.4 is considered statistically significant 20. Cross-validation (CV)-ANOVA p-values for each model were determined and RMSECV (root mean square error of cross-validation) values calculated.

To test the predictive ability of key models, independent testing sets of spectra obtained from additional animals (Figure 1) were produced and classified in a blinded fashion. Results are presented as 2x2 contingency tables and ROC curves with Fisher's exact statistics.

### Metabolite identification and quantification

The variables deemed to have the greatest impact driving the separations were those with a variable importance in projection (VIP) score > 2.0. Comparison of important bucket integrals by ANOVA followed by Dunnett's multiple comparison tests allowed determination of direction, magnitude and significance of metabolite changes between groups. Peaks present in important buckets were identified using a combination of COSY NMR, literature values and reference to the human metabolome database 21-24. Relative quantitation of specific metabolites was performed by summing the integral regions of each metabolite. Significances of changes were determined by 1-way ANOVA followed by Dunnet's multiple comparison post-hoc tests comparing each time-point.

## Results

### Intracerebral tumour models

**Figure 2:**
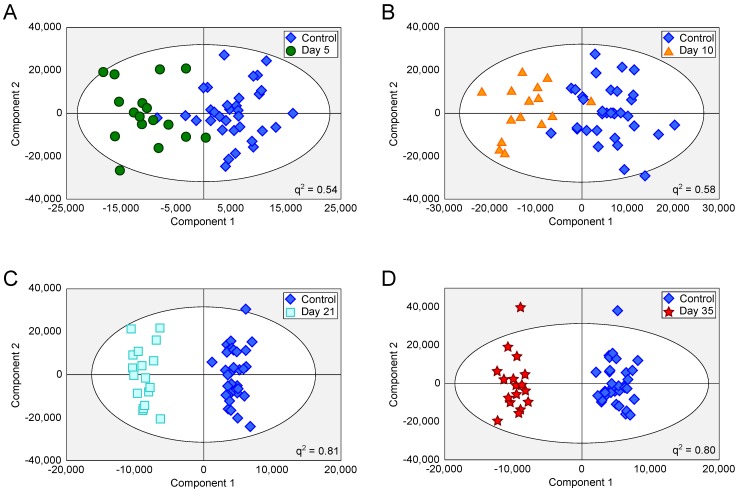
** OPLS-DA scores scatter plots for intracerebral 4T1-GFP models.** OPLS-DA scores scatter plots for models constructed to separate urine samples from animals in control cohorts from animals at day 5 (A), 10 (B), 21 (C) and 35 (D) after intracerebral injection of 4T1-GFP cells. For days 5, 10, 21 and 35, q^2^ values were 0.54, 0.58, 0.81 and 0.80, respectively, whilst CV-ANOVA p-values were 3.0x10^-7^, 6.1x10^-6^, 6.7x10^-8^ and 1.5x10^-8^, respectively; q^2^ values > 0.4 are considered biologically significant. RMSECV values were 0.32, 0.30, 0.20 and 0.21 respectively.

**Figure 3:**
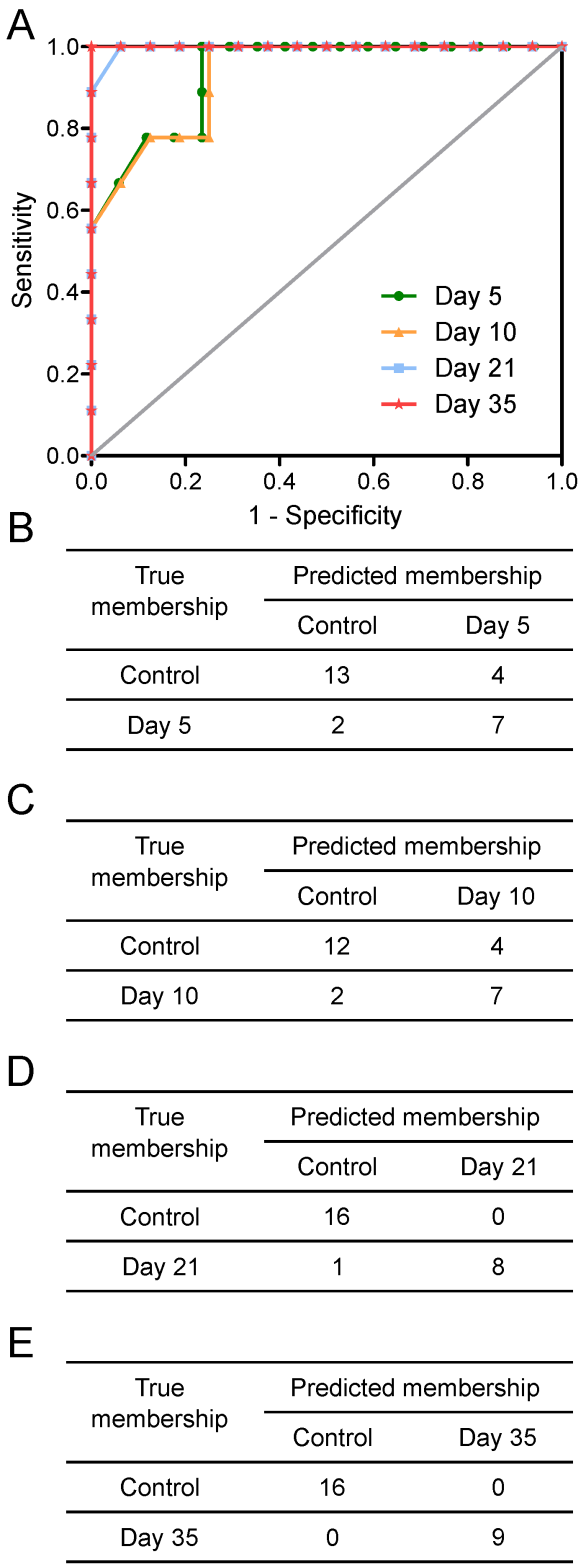
** Prediction results.** Prediction results from OPLS-DA models separating animals at different time points after intracerebral injection of 4T1-GFP cells represented as (A) ROC curves and (B to E) 2x2 contingency tables for days 5, 10, 21 and 35.

Tumours injected intracerebrally grew as described previously 10, reaching maximum diameters of 400-500µm at day 5 and 500-600µm at day 10. Tumours were not visible by current best clinical practice Gd-enhanced MRI at either day 5 or 10. By day 21, faint Gd-enhancement was visible at most sites of tumour induction and by day 35 frank breakdown, as seen clinically, was observed in all cases. For the day 10 timepoint, with three different cell line tumours (Table 1), tumour volumes were estimated by histology for representative animals. Tumour volumes were 100±90nL, 30±11nL and 62±34nL for the B16F10, MDA231BR and 4T1-GFP tumours respectively. There was no significant difference in tumour volumes between groups (p>0.05; one-way ANOVA followed by Tukey's post hoc test). Urine NMR spectra were acquired (e.g. Figure S1 and Figure S2) and OPLS-DA models constructed separating samples from 5, 10, 21 or 35 days after 4T1-GFP cell injection from their respective control cohorts (see Figure 1 for study schematic). All models were significantly predictive, with models from later time-points being more predictive than those from earlier time-points (Figure 2 and Movie S1). Model predictive ability was tested using the withheld testing set. Unknown samples were assigned to control or 4T1-GFP groups and contingency tables and ROC curves constructed (Figure 3). Models had high sensitivity and specificity: 0.78 and 0.76 at day 5; 0.78 and 0.75 at day 10; 0.89 and 1.00 at day 21; and 1.00 and 1.00 at day 35, respectively. Progression of the testing set is shown in Movie S2. As for the 4T1-GFP model, both the B16F10 and MDA231BR-GFP cohorts yielded significant separations between the tumour-injected and combined control cohorts (Figure S3).

In the 4T1-GFP cohort, eight metabolites were identified from the VIP plots as contributing the most to the separation of some or all models. These metabolites were allantoin (δ=5.38-5.39), citrate (δ=2.52-2.53), trimethylamine (TMA, δ=3.28-3.29), trimethylamine-*N*-oxide (TMAO, δ=3.27-3.28), 2-oxoglutarate (δ=2.43-2.45), creatinine (δ=4.06-4.07), taurine (δ=3.27-3.44) and creatine+phosphocreatine (Cr+PCr - indistinguishable by ^1^H NMR at this pH; δ=3.93-3.94). Where metabolite peaks span a number of buckets, only the most important buckets are listed.

**Figure 4:**
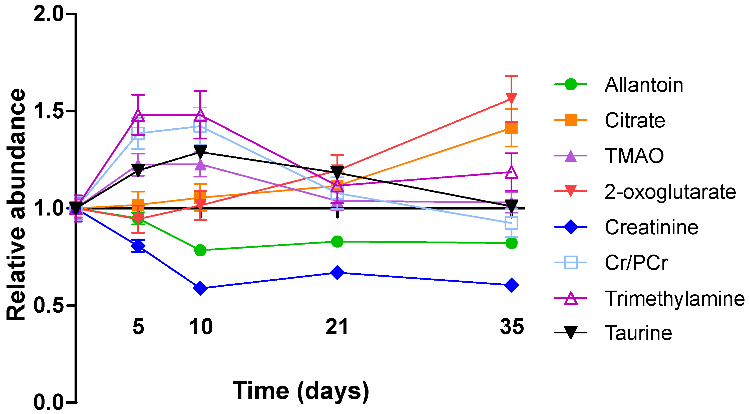
** Metabolite abundance timecourse. **Metabolite concentration changes throughout the 4T1-GFP metastasis timecourse. Abundances are shown relative to that found in each control population. Data points are shown as mean ±SEM. *n*=17 for each timepoint.

TMA, TMAO, Cr+PCr and taurine were all more abundant in the early stages of tumour development with their importance for model separation decreasing by day 21 (all p>0.05 at days 21 and 35; Figure 4). TMA was increased 48% and TMAO was increased 23% at both days 5 and 10 (p<0.01). Cr+PCr was increased 39% at day 5 (p<0.01) and 42% at day 10 (p<0.001). Taurine was increased significantly only at day 10 (+29%, p<0.01). Neither citrate nor 2-oxoglutarate changed at days 5 or 10, but increased gradually across the time-course to reach significance by day 35: (citrate +42% and 2-oxoglutarate +57%; p<0.001).

Creatinine and allantoin both decreased significantly from day 0 to 10 (Figure 4). Creatinine abundance relative to control was 81% at day 5; 59% at day 10; 67% at day 21 and 61% at day 35 (all p<0.001). Allantoin abundance relative to control was unchanged at day 5 (95%), but decreased significantly thereafter: 79% at day 10; 83% at day 21; 82% at day 35 (all p<0.001).

Metabolite changes in the alternative cell line models showed some similarities to those observed in the 4T1-GFP models. The greatest similarity was the profile of creatinine and Cr+PCr, which were mirrored in both of the breast carcinoma metastasis models (4T1-GFP and MDA231BR-GFP). Interestingly, notable differences were evident between the breast carcinoma and melanoma brain metastasis models; e.g. citric acid was unchanged in the 4T1-GFP and MDA231BR-GFP models at day 10, but was decreased in the B16F10 model at the same time point. For reference, proton NMR spectra of cell suspensions for each of the cell lines used are included in Figure S4.

### Differing CNS and systemic tumour burdens

**Figure 5:**
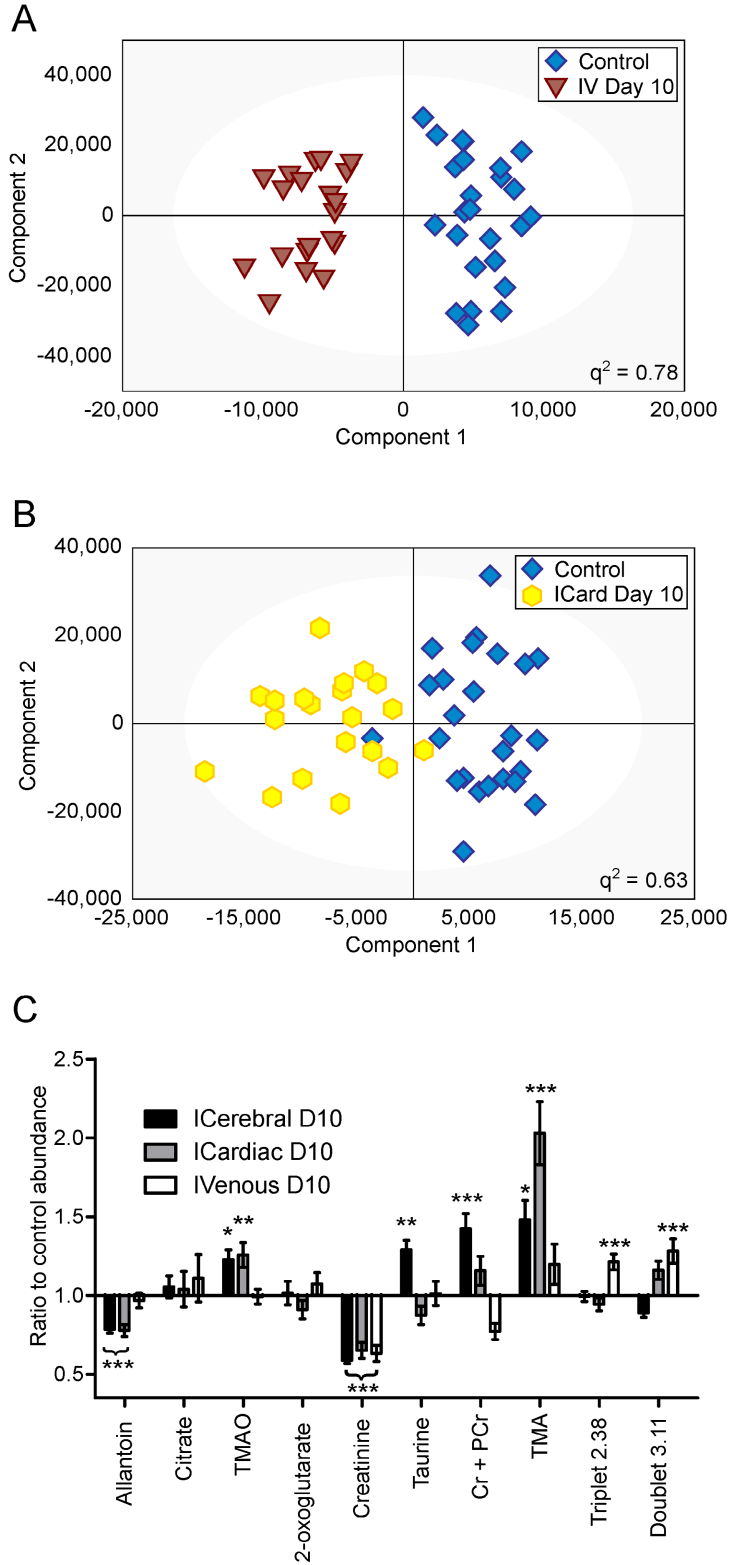
** Differing injection route models. **OPLS-DA scores scatter plots for models constructed to separate urine samples from animals in control cohorts from animals at day 10 after injection of 4T1-GFP tumour cells either (A) intravenously or (B) intracardially. q^2^ values were 0.78 and 0.63 for intravenous and intracardiac routes, CV-ANOVA p-values were 4.7x10^-8^ and 8.8x10^-4^, and RMSECV values were 0.23 and 0.30, respectively. (C) Relative abundances of key metabolites in 4T1-GFP brain metastasis models with tumour induction by differing routes. Each metabolite's abundance is shown relative to each group's control cohort abundance at day 10 after injection. Intracerebral injections are shown with black bars (n=50), intracardiac injections are shown with grey bars (n=44) and intravenous injections are shown with open bars (n=44). Data are means ± SEM. * = p<0.05; ** = p<0.01; *** = p<0.001.

OPLS-DA models separating control cohorts from animals injected with 4T1-GFP cells via the intravenous or intracardiac routes were significantly predictive at day 10 (Figure 5A and B). A comparison was made between metabolites contributing to separations in the intracardiac, intravenous and intracerebral 4T1-GFP models at day 10 (Figure 5C). Only the eight metabolites identified as contributing most strongly to the time-course of tumour progression in the intracerebral model were considered.

Creatinine was decreased by approximately the same amount for all three routes of induction (59, 65 and 63% control in intracerebral, intracardiac and intravenous models, respectively; p<0.001). Allantoin, TMA and TMAO were unchanged in the intravenous model, but allantoin was decreased in the intracardiac model (78% control; p<0.001) whilst TMA and TMAO were increased (203 and 123% control; p<0.001 and p<0.01, respectively), in line with the changes seen in the intracerebral model. Cr+PCr was only significantly increased in the intracerebral model (142% control; p<0.001); neither the increase in Cr+PCr in the intracardiac model (116% control), nor the decrease in the intravenous model (77% control), reached significance. Despite being increased in the intracerebral model (129% control; p<0.01), taurine was not changed significantly in the intracardiac or intravenous models. No change in abundance of either citrate or 2-oxoglutarate was observed in any model at day 10; these two metabolites only changed significantly at later time-points in the intracerebral time-course (Figure 4). In addition to these metabolites, an unidentified triplet centred at δ=2.38 and an unidentified doublet at δ=3.11 were both increased in the intravenous model (121 and 128% control respectively; p<0.001), but showed no significant changes in either the intracerebral or intracardiac models.

## Discussion

We have shown that we can reliably separate mice with focal brain metastases from controls as early as five days after tumour induction, using urine ^1^H NMR analysis and multivariate statistical pattern recognition. We also separated control animals from those injected with 4T1-GFP cells via either the intracardiac or intravenous routes, each giving rise to differing systemic or CNS metastatic burdens. Further validation cohorts have demonstrated this approach has utility in tumours from multiple primary cell types. Together, these findings suggest that this biofluid-based metabolomics approach could have considerable utility for detecting brain metastases earlier than current clinical approaches.

### Metastatic models

Owing to careful experimental design of the control cohorts, we can be sure that the observed separations between metastasis-bearing and control animals are independent of any urinary changes induced by the intracerebral injection, aging or hormonal cycles. The ROC analysis and prediction results further show that the urinary metabolic profile, as determined by ^1^H NMR, is sufficient to sensitively and specifically distinguish animals with tumours from animals without.

Currently, it is possible to determine the presence of systemic metastases by either biopsy 25 or the use of specific blood markers 26, but the same is not true for brain metastases. The ability to detect intracerebrally injected 4T1-GFP cells at an early time-point suggested that it may be possible to identify a set of metabolites specific to brain metastases, even in the presence of systemic metastases. The 4T1-GFP mouse mammary carcinoma cell line 27 has been widely used as a model of metastasis. By varying the injection route, it is possible to bias the sites of resulting metastases. For example, intravenous tumour cell injections give rise to metastatic nodules primarily in the lungs, as this is the first capillary bed encountered. Intravenous injection does not give rise to any metastases in the brains of mice. Alternatively, intracardiac injections put tumour cells directly into the arterial circulation which gives rise to metastases in a wider range of locations including brain, bone, liver, lung and adrenal glands 10,15,28. Thus, comparing commonalities between intracardiac injection and direct injection of cells into the brain, and differences from intravenous cell injection, we may identify a fingerprint for brain metastasis specifically.

The pattern of key metabolite abundance changes for the differing routes of 4T1-GFP cell injection indicates that systemic and CNS metastases may give rise to different urinary metabolic profiles, which may be super-imposable where metastases exist in both locations. For example, the decrease in allantoin observed at the day 10 time-point for both the intracerebral and intracardiac injections is of particular note, since it was unchanged in the intravenous model. At the same time, Cr+PCr and the unidentified doublet at 3.12ppm showed opposite responses in the intravenous and intracerebral models, whilst the intracardiac model lay between the two extremes. This arrangement may reflect a combination of systemic and intracerebral metastatic phenotypes in the intracardiac model. Taken together, these observations support the hypothesis that there is a metabolic fingerprint that is specific to animals with a brain metastasis burden, and is outwith any changes induced by systemic metastases. In part, these fingerprints have strong contributions from specific metabolites as identified and discussed, but it is important to note that the entire urine NMR spectrum contributes to each model. Thus, it is unlikely that a simple diagnostic of the strongest correlating metabolites will have the same predictive power as a model constructed from complete spectral data. Nevertheless, throughout all of the models investigated the abundance of allantoin has been one of the most prominent contributors to the success of models that identify animals with brain metastases. Since human metabolism stops before allantoin, at uric acid, an NMR-invisible compound, it may be warranted to assay uric acid independently in human urine to supplement the data available from ^1^H NMR spectroscopy.

### Model sensitivity to micrometastases

In this study intracerebrally injected metastatic colonies are still very small, 400-500µm in diameter at day 5 and 500-600µm in diameter at day 10. If these tumours were spherical, this would correspond to tumour volumes of 34-65nL and 65-110nL, respectively. However, the tumours do not grow in perfect spheres and the actual tumour volumes observed at day 10 demonstrate that whilst the B16F10 tumours are roughly spherical, the diameter of the MDA231BR-GFP and 4T1-GFP tumours, which grow in a much more stellate pattern, also include normal brain tissue. The minimum size of metastasis currently detectable in the clinic, using the current best practice Gd-enhanced MRI, is *ca *0.5cm in diameter (volume 65µL), but a more realistic detection diameter is 1cm (volume 520µL). Thus, the micrometastatic foci detectable here are 3-4 orders of magnitude smaller than the current clinical threshold.

The intracardiac model, also at day 10, produces metastases in the brain that are typically 100-200µm in diameter (a volume of around 0.5-4.2nL). These very small micrometastases are spread throughout the brain in a diffuse manner at a density of around 0.2 tumours/mm^2^ brain section 10. Thus, the finding of a positive separation from the control cohort in this model, further indicates the sensitivity of this approach for the diagnosis of micrometastases.

We have considered the possibility that these classifications represent a general “neuroinflammatory” phenotype, rather than a specific brain tumour phenotype. Previous work by our group studying multiple sclerosis (MS) patients produced significant and predictive models separating relapsing-remitting from secondary progressive MS, as well as from controls 12. In the same study, neurological control cohorts of Alzheimer's disease patients and amyotrophic lateral sclerosis patients failed to separate from their respective clinic's normal control cohort, indicating in that study that there was a distinct separation for MS and not a general separation for neurological disease with an inflammatory component. Some of the stronger metabolic changes observed in the MS serum models were in glucose and fatty acids, metabolites not found in urine. However, our pilot models with blood from mice with metastases did not show strong separations (data not shown). Moreover, previous work by our group studying metabolic profiles in rat urine 29 showed distinct metabolic profiles after lesions were induced in the brain using TNFα or IL-1β. In that study, the distinct profiles again indicated that there were specific phenotypes from different stimuli, not a generic neuroinflammation phenotype. Finally, whilst some metabolites in that study also changed in the current study (e.g. citrate and 2-oxoglutarate), many others remain unchanged herein (e.g. leucine, isoleucine and valine) adding further evidence to our hypothesis that distinct metabolic phenotypes arise from different physiological sources.

### Clinical translation

Clinical translation of this technique is likely to prove useful for screening patients at risk of brain metastasis. In this setting, knowing the exact constituents of metabolic profiles is less important than being able to compare patients to groups with known diagnoses. For example, patients are easily stratified by primary tumour (e.g. melanoma or breast carcinoma) so could be assessed using a model constructed from only patients with that type of primary.

Urine samples are easy and cheap to collect, and NMR analysis is inexpensive. We envisage a routine screening of patients in follow-up clinics where samples are analysed and plotted in a dynamic fashion against gold standard models produced from patients with confirmed diagnoses. These dynamic plots would be similar to that shown in Movie S2; symbol movement over time is analogous to the progression we would expect from repeated patient samples at multiple clinic visits. The reference model allows us to define probability cut-offs, which in turn allow clinicians to classify patients as having, or not having, clinically occult brain metastases. Since we have shown detection at the micrometastatic stage in our models, we anticipate that detection of currently occult metastases would become possible in human patients.

## Summary

Diagnosis of patients with brain metastases at an early time-point remains a clinically intractable problem. Urinary metabolomics has been shown to be a powerful screening approach to separate diseased individuals from controls, e.g. in Barrett's oesophagus and oesophageal carcinoma 30, or in separating oral squamous cell carcinomas from oral leukoplakia patients and control groups 31. However, neither of those studies included a separate validation cohort, leaving open the possibility of co-incidence in the models generated. Here, we have shown that models based upon complete ^1^H NMR urine spectra, with contributions from all urinary metabolites, can be used to sensitively and specifically predict presence of brain metastases in mice. On this basis, we believe that human validation studies are now warranted.

## Supplementary Material

Additional File 1 Figures S1-S4.

Additional File 2 Movie S1.

Additional File 3 Movie S2.

## Figures and Tables

**Table 1:** Summary of animal models used in this paper.

Cell type and animals	Route of injection	Post-injection analysis points (days)
4T1 murine mammary carcinmoa cells in female BALB/c mice	Intracerebral	5, 10, 21 and 35
	Intracardiac	10
	Intravenous	10
MDA-231BR human mammary carcinoma cells in female SCID mice	Intracerebral	10
B16F10 murine melanoma cells in BL/6 mice	Intracerebral	10

## Abbreviations

BBB: Blood-brain barrier
CNS: Central nervous system
GFP: Green fluorescent protein
NMR: Nuclear magnetic resonance
OPLS-DA: Orthogonal partial least-squares discriminant analysis
ROC: Receiver operator characteristic.
