# Supplementary material for: Early Diagnosis of Brain Metastases Using a Biofluids-Metabolomics Approach in Mice
Source: Theranostics. 2016 Sep 12;6(12):2161–9. doi: 10.7150/thno.16538 (PMC5135440; doi:10.7150/thno.16538)
Supplement: Additional File 1 — Figures S1-S4. [file thnov06p2161s1.pdf]

Movie S1: Animated progression of individual animals in control (blue circles) and tumour-bearing (green squares) groups in the 4T1-GFP intracerebral metastasis model. Positions at days 5, 10, 21 and 35 are set based upon the respective OPLS-DA models and movements are interpolated linearly between these points.

Movie S2: Animated progression of the blinded testing set (moving, intensely colour symbols), as predicted by the D35 working set (faint, static symbols). Control samples are blue circles and tumour-bearing animals are in green squares. Sample positions at day 0, 5, 10, 21 and 35 are based on predicted positions by the day 35 model. Movements are interpolated linearly between these points.

Figure S1: Example urine NMR spectrum with peaks of interest annotated. PCr = Phosphocreatine. TSP = 3-trimethylsilyl-1-[2,2,3,3,-$^{2}H_4$] propionate.

Figure S2: Representative spectra from one animal as tumour growth progresses throughout the 35 day timecourse.

Figure S3: OPLS-DA plots of urine NMR spectra separation in (A) C57/BL6 mice from the B16F10 cell cohort vs. the respective combined PBS and naïve control cohort and (B) SCID mice from the MDA-231-BR-GFP cell cohort vs. the respective combined PBS and naïve control cohort.

Figure S4: ^{1}H NOESY presat spectra of intact cell suspensions from each of the three cell lines used

Figure S1

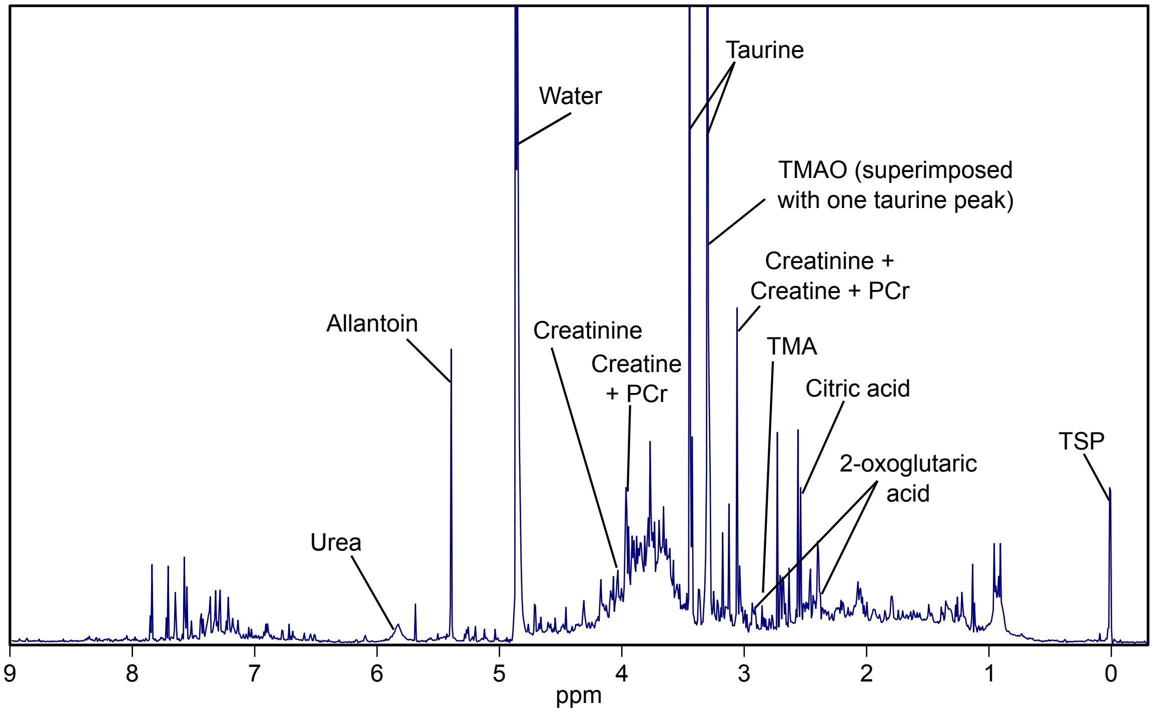
Water
Taurine
TMAO (superimposed with one taurine peak)
Creatinine + Creatine + PCr
Allantoin
Creatinine
Creatine + PCr
TMA
Citric acid
2-oxoglutaric acid
TSP
Urea
9
8
7
6
5
4
3
2
1
0
ppm

Figure S2

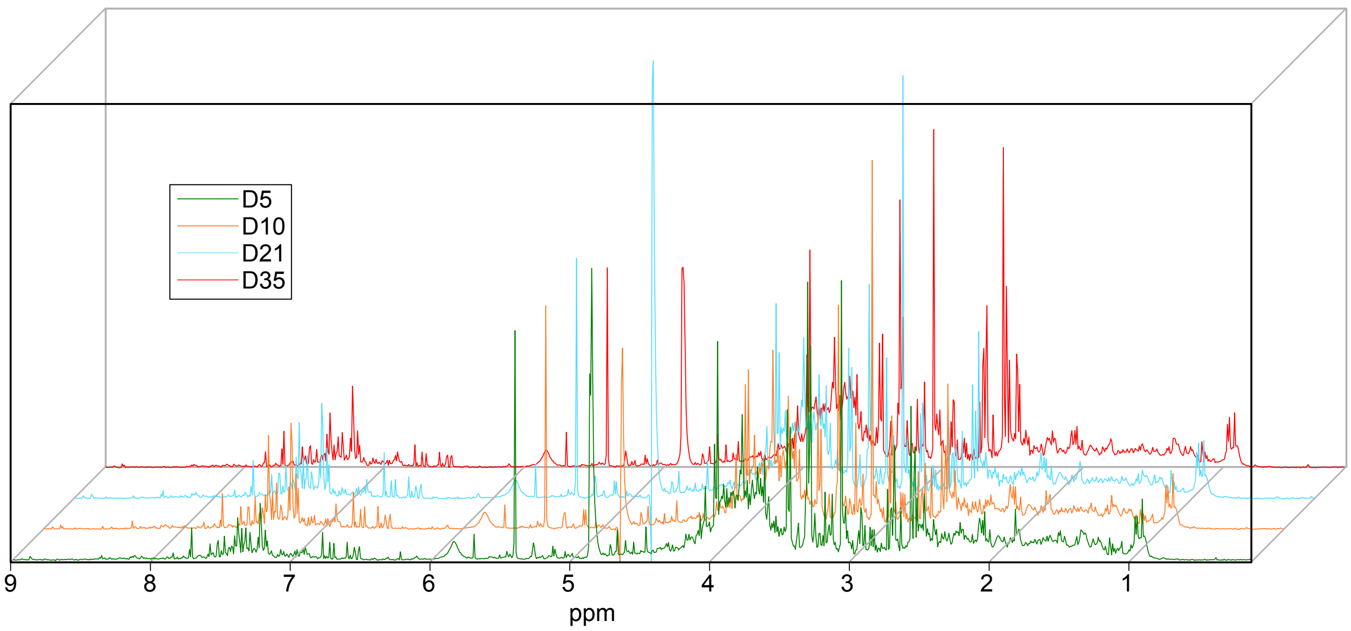

Figure S3

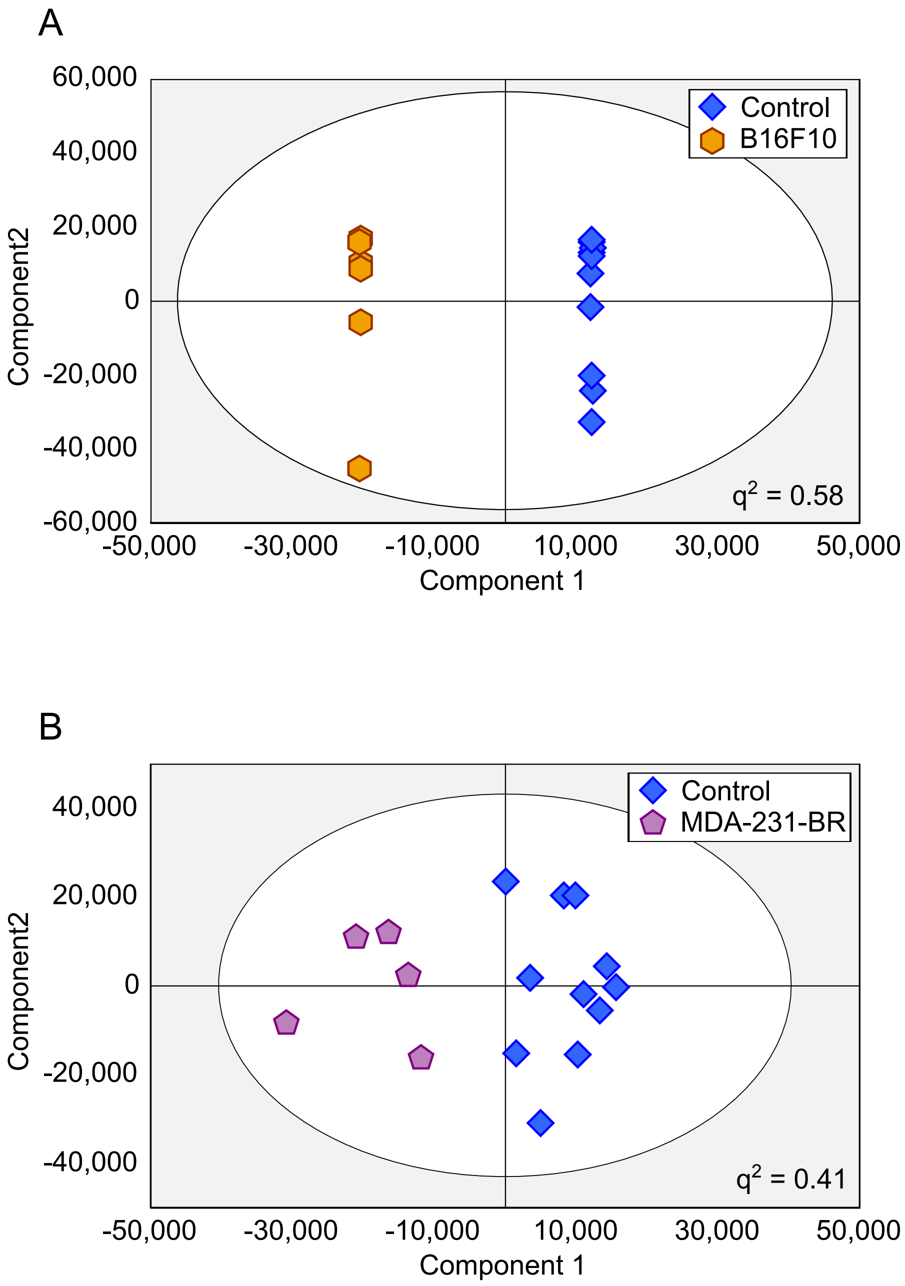
A
60,000
40,000
20,000
0
-20,000
-40,000
-60,000
Component2
-50,000
-30,000
-10,000
10,000
30,000
50,000
Component 1
Control
B16F10
q2 = 0.58
B
40,000
20,000
0
-20,000
-40,000
Component2
-50,000
-30,000
-10,000
10,000
30,000
50,000
Component 1
Control
MDA-231-BR
q2 = 0.41

Figure S4

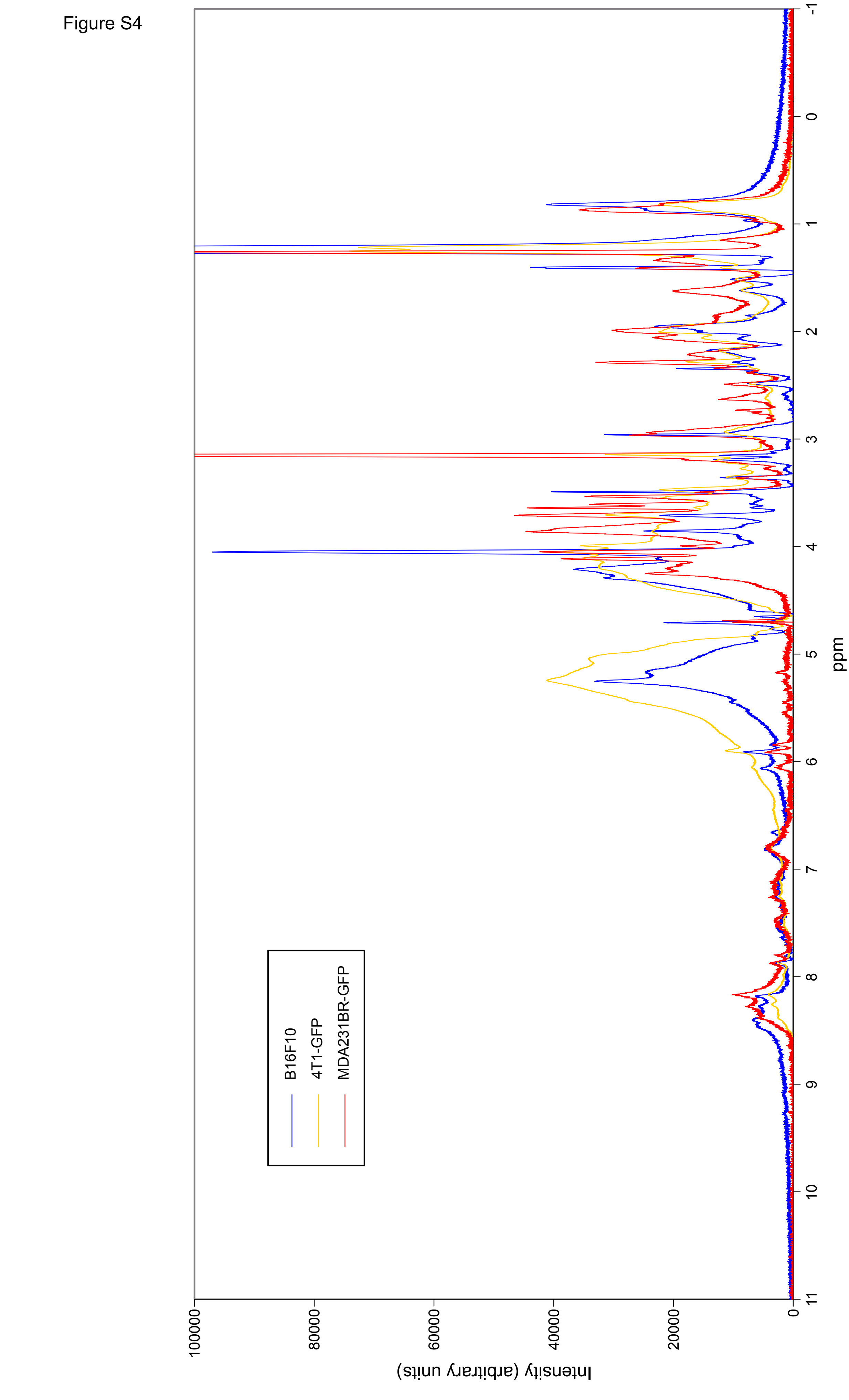
Intensity (arbitrary units)
ppm
B16F10
4T1-GFP
MDA231BR-GFP
0
20000
40000
60000
80000
100000
11
10
9
8
7
6
5
4
3
2
1
0
-1
